# Reimagining paediatric care: technology, trust, and the global movement for child-centred innovation

**DOI:** 10.3389/fmed.2026.1782611

**Published:** 2026-06-30

**Authors:** Paul Dimitri

**Affiliations:** 1 University of Sheffield, Sheffield, United Kingdom; 2 Sheffield Children’s NHS Foundation Trust, Sheffield, United Kingdom

**Keywords:** children, digital, innovation, paediatrics, technology

## Abstract

Technology is rapidly reshaping paediatric healthcare, offering unprecedented opportunities to improve outcomes, personalise care, and extend reach beyond traditional clinical settings. From AI-driven diagnostics and wearable monitoring to immersive therapeutics and digital mental health tools, innovation is enabling more proactive, child-centred models of care. In the UK, initiatives such as the National Centre for Child Health Technology (NCCHT) and the NIHR HealthTech Research Centre in Paediatrics and Child Health are driving strategic adoption, while international networks including, KidsUp in the US, EPTRI, i4Kids in Spain, ISPI, the WHO’s Global Digital Health Strategy and EU-funded paediatric innovation consortia are fostering cross-border collaboration and knowledge exchange. Despite this momentum, significant challenges remain. Fragmented infrastructure, limited interoperability, and uneven digital literacy across the workforce hinder widespread implementation. Regulatory frameworks often lag behind technological advances, particularly in areas like AI, where transparency, bias mitigation, and safeguarding are critical. Moreover, funding pathways for paediatric-specific technologies remain underdeveloped compared to adult-focused innovation. Children and young people (CYP) are increasingly vocal about their expectations for health tech. They value tools that are intuitive, inclusive, and respectful of their autonomy. Feedback from CYP engagement exercises highlights a desire for technologies that support mental wellbeing, facilitate communication with clinicians, and offer personalised insights, without feeling intrusive or overtly clinical. However, concerns persist around data privacy, digital exclusion, and the potential for technology to replace human connection. Ethical considerations are also central to paediatric digital health. AI applications must be transparent, accountable, and co-designed with children and families to ensure they reflect lived experience and avoid unintended harm. Equity must be embedded from the outset, ensuring that innovation does not widen disparities in access, outcomes, or trust. To realise the full potential of technology in paediatrics, we must build inclusive, ethically grounded ecosystems that centre children’s voices, support the workforce, and enable safe, scalable innovation. This requires sustained investment, cross-sector collaboration, and a commitment to embedding digital transformation within the broader goals of child health equity and empowerment.

## 1 Introduction

Technology and digital transformation is reshaping the foundations of paediatric healthcare. Rapid advances in science and technology have resulted in CYP with chronic and rare conditions now surviving well into adulthood (1). Therefore, technological developments must start early and meet the needs of CYP to ensure better health and prosperity in adulthood. Over the past decade, rapid advances in artificial intelligence (AI), machine learning (ML), wearable biosensing, digital computing, and precision medicine have created unprecedented opportunities to shift paediatrics toward proactive, predictive, personalised, and continuous models of support (2, 3). These technologies offer the potential to address longstanding challenges in child health, including diagnostic delays, limited specialist availability, fragmented care pathways, and the rising burden of chronic physical and mental health conditions among children and young people (CYP). Importantly, when developing digital and AI technologies, dynamic changes in cognition, and psychosocial development, age-specific physiological and anatomical changes, and the vulnerabilities and rights of CYP need to be addressed (4).

Importantly, investment in child health is not only a moral and clinical imperative but also an economic one. Evidence demonstrates that countries prioritising the health of CYP achieve significant financial returns, with each pound (£) spent on child health generating more than £10 in societal value over the course of a lifetime (5, 6). This return reflects reduced healthcare costs, increased productivity, and enhanced contributions to society as healthier individuals grow into adulthood. Moreover, the global paediatric healthcare market was valued at approximately USD 11.9 billion in 2018 and was expected to generate around USD 16 billion by 2025 (7). However, despite the transformative potential of new technologies, paediatric innovation has historically been constrained by structural underinvestment, small and heterogeneous populations, and regulatory frameworks that were not designed with CYP in mind (8, 9). Fewer than 5% of medical devices are developed specifically for paediatric use, and many digital tools have been adapted from adult technologies without adequate validation across developmental stages. In response, several national and international initiatives have emerged to strengthen the paediatric innovation ecosystem. In the United Kingdom, the National Centre for Child Health Technology (NCCHT) (10) and the NIHR (National Institute of Health and Care Research) HealthTech Research Centre (HRC) in Paediatrics and Child Health (formally known as the NIHR Children and Young People MedTech Cooperative (NIHR CYP MedTech)) (11) have been established as infrastructures to address the unmet healthcare needs of CYP through technology prototyping, clinical evaluation and supporting adoption. Globally, networks such as KidsUp in the United States, the European Paediatric Translational Research Infrastructure (EPTRI), i4Kids in Spain, and multiple EU-funded consortia are fostering cross-border collaboration, harmonising standards, and accelerating the development of child-centred technologies (12–15).

Yet significant barriers continue to impede progress. Digital infrastructure remains fragmented, with limited interoperability between electronic health records, community systems, and emerging data streams from wearables and home-based sensors. Workforce digital literacy varies widely, and clinicians often lack the training, confidence, or time required to integrate digital tools into routine practice. Regulatory frameworks struggle to keep pace with rapidly evolving technologies, particularly in relation to adaptive and transformative AI. Ethical concerns remain at the forefront of paediatric technology development including data privacy, algorithmic bias, digital exclusion, and safeguarding, where children’s rights, developmental needs, and vulnerabilities must be central to design, deployment and the subsequent process of assessment and reassessment of technologies in real-life and in real-world clinical settings (16). Moreover, CYP are increasingly shaping the digital health agenda. CYP consistently express that they want health technologies to be easy to use, personalised, and supportive of their independence. CYP value tools that fit seamlessly into their daily lives, helping them manage long-term conditions without adding extra complexity. They want reassurance that their privacy and data are protected, and they want professionals to remain involved, so that they feel supported rather than left alone with technology. Above all, they want to be part of the design process, ensuring that the tools reflect their real needs and experiences. These perspectives underscore the need for co-design, participatory governance, and rights-based approaches to digital health innovation (17, 18).

By examining both the opportunities followed by the systemic challenges that shape this rapidly evolving field in paediatrics and child health, this article aims to provide insight for policymakers, clinicians, researchers, and technologists who are seeking to build trustworthy, scalable, and ethically grounded ecosystems for paediatric innovation.

## 2 Infrastructure to support child health technology development

The need for dedicated infrastructures for child health technology reflects a growing global movement toward child-centred innovation, recognising that CYP have unique healthcare and safeguarding needs that cannot be met by systems designed fundamentally for adult health and healthcare. Investment in specialised centres and international networks provides the capacity to prototype, clinically evaluate, and accelerate the adoption of technologies tailored to paediatric populations. This infrastructure is critical not only for addressing unmet clinical needs but also for embedding the voices of CYP and their families into the innovation process, ensuring that solutions are relevant, acceptable, and equitable. Globally, several infrastructures have been established to foster cross-border collaboration, harmonisation of standards, and acceleration of innovation in child health technology. The United Kingdom has established a robust infrastructure to accelerate the development, evaluation, and adoption of child health technologies. Central to this effort is the development of the NCCHT, based at Sheffield Olympic Legacy Park, England UK, which will provide a dedicated environment for prototyping, clinical validation, and translational research in paediatric health innovation when it opens in 2026. The NCCHT represents a unique collaboration between healthcare providers, academia, and industry, aiming to address unmet needs in diagnostics, therapeutics, and digital health for CYP, with the aim of producing the most advanced technological healthcare system in the world (10). Complementing this initiative, the NIHR HRC in Paediatrics and Child Health (UK), launched in 2024, builds upon the legacy of the NIHR Children and Young People MedTech Cooperative. This HRC is the only NIHR HRC (of which there are 14) dedicated exclusively to CYP, providing national leadership in the evaluation of medical devices, digital platforms, and assistive technologies. Its remit includes supporting early-stage innovation, generating evidence for adoption within the NHS, and embedding patient and family involvement throughout the innovation cycle (11). Over its lifespan, NIHR CYP MedTech achieved significant impact by securing £17.1 million for innovation projects and catalysing a further £27 million generated by industry partners. It delivered 180 collaborative projects spanning healthcare, academia, and industry, and actively supported knowledge exchange through 68 conferences and events. The programme engaged extensively with the innovation ecosystem, working alongside 139 SMEs and 44 global companies, thereby strengthening translational pathways for child health technologies. A landmark achievement was the launch of the UK’s Child Health Technology Conference in 2021, which has since become a national and international platform for showcasing advances in paediatric innovation and fostering multidisciplinary collaboration. GOSH DRIVE and Alder Hey Innovation are leading UK hospital-based hubs that embed digital and medtech innovation directly into paediatric care. At Great Ormond Street Hospital (GOSH), the Data Research, Innovation and Virtual Environments (DRIVE) unit functions as a state-of-the-art centre for digital innovation (19, 20). Established in 2018, DRIVE aims to transform the use of data and technology in healthcare by supporting the development, early-stage testing, and scaling of solutions across the NHS. It has facilitated more than 300 projects, ranging from advanced data dashboards to AI-enabled clinical tools, and provides an innovation hub whereby staff, industry, and academia collaborate to co-design technologies that improve outcomes and patient experience for children and young people. Alder Hey Children’s Hospital in Liverpool, UK has created Alder Hey Innovation, a hospital-led innovation hub integrating digital medicine, and artificial intelligence to solve real-world paediatric healthcare challenges (21). Recent investment of over £4 million into the Liverpool Paediatric Open Innovation Zone (POIZ) (22) has further strengthened its role as a national leader, enabling collaborative projects with industry and academia to deliver cutting-edge solutions such as remote cardiac monitoring platforms and personalised digital health tools.

In the United States, the KidsX Accelerator provides a structured platform for start-ups and health systems to co-develop paediatric digital health solutions, with a strong emphasis on scalability, clinical integration, and rapid adoption into healthcare pathways. By linking innovators directly with children’s hospitals, KidsX ensures that technologies are tested in real-world paediatric settings, thereby reducing the translational gap between prototype and clinical practice (12). In Europe, EPTRI has created a pan-European network designed to support paediatric drug and device development. EPTRI offers shared resources for preclinical and translational research, including biobanking, genomics, and advanced modelling platforms. Its mission is to overcome the fragmentation of paediatric research by providing a coordinated infrastructure that enables harmonised methodologies, regulatory alignment, and equitable access to innovation across member states (13). The i4Kids Pediatric Innovation Hub, coordinated by Sant Joan de Déu Barcelona Children’s Hospital in Spain integrates hospitals, universities, and industry partners to accelerate the development of child-centred technologies. By fostering multidisciplinary collaboration, i4Kids supports the creation of medical devices, digital platforms, and therapeutic innovations specifically tailored to the needs of CYP (14). In addition, multiple EU-funded consortia have been established to strengthen the paediatric innovation ecosystem. The c4c network provides a pan-European platform for multinational paediatric clinical trials including medical device trials, enabling standardised protocols and regulatory alignment across borders (15). Similarly, ADD4KIDS focuses on accelerating demand-driven tools for paediatric innovation adoption, ensuring that technologies are not only developed but also integrated into healthcare systems (16) (Table 1).

**Table 1:** Global infrastructures supporting child-health technology innovation.

Region/Country	Infrastructure/Organisation	Description	Core functions and impact
United Kingdom	National Centre for Child Health Technology (NCCHT)	A world-leading centre opening in 2026 at Sheffield Olympic Legacy Park, dedicated to prototyping, clinical validation, and translational research in paediatric technologies.	Drives diagnostics, therapeutics, and digital innovation; integrates healthcare, academia, and industry; aims to create the most advanced child-health technology ecosystem globally.
United Kingdom	NIHR HealthTech Research Centre (HRC) in Paediatrics and Child Health	The only NIHR HRC dedicated exclusively to CYP, launched in 2024, building on NIHR CYP MedTech.	National network in device evaluation, digital platforms, assistive tech; supports early-stage innovation; embeds CYP and family involvement.
United Kingdom	GOSH DRIVE	A digital innovation hub at Great Ormond Street Hospital, established 2018.	Delivered >300 projects; accelerates AI tools, data dashboards, virtual environments; embeds co-design with clinicians and families.
United Kingdom	Alder Hey Innovation	Hospital-based innovation hub integrating digital medicine and AI.	£4 M Liverpool POIZ investment; advances remote monitoring, personalised digital tools, and industry–academic collaboration.
United States/International	KidsX Accelerator	A leading international paediatric digital-health accelerator linking start-ups and larger industry partners with children’s hospitals.	Supports rapid prototyping, real-world testing, and scalable adoption across US and international paediatric systems.
Europe (Pan-EU)	EPTRI – European Paediatric Translational Research Infrastructure	A pan-European network supporting paediatric drug, device, and digital innovation.	Provides biobanking, genomics, modelling platforms; harmonises standards; reduces fragmentation in paediatric research.
Spain / Europe	i4Kids Pediatric Innovation Hub	Coordinated by Sant Joan de Déu Barcelona Children’s Hospital to accelerate the development and adoption of child health technologies across Europe.	Integrates hospitals, universities, and industry; accelerates device development, digital platforms, and therapeutic innovation.
Europe (Pan-EU)	c4c – conect4children	EU-wide platform for multinational paediatric clinical trials.	Standardises protocols, aligns regulatory processes, and strengthens trial capacity for drugs and devices.
Europe (Pan-EU)	ADD4KIDS	EU consortium focused on demand-driven paediatric innovation.	Supports adoption pathways, system integration, and cross-border collaboration for child-health technologies.
Global	International Paediatric Innovation Networks	Emerging alliances linking hospitals, research institutes, and industry worldwide.	Promote harmonisation of standards, shared evaluation frameworks, and global knowledge exchange.

A summary of major international centres, networks, and platforms dedicated to accelerating paediatric technology development, evaluation, and adoption.

These infrastructures collectively form a global ecosystem for child-centred innovation and technology development, ensuring that technologies are rigorously tested, ethically implemented, and equitably accessible. They embed patient and family involvement, harmonise regulatory standards, and foster collaboration across healthcare, academia, and industry, positioning paediatric innovation as a driver of improved outcomes and long-term health. Moving forward, one of the major challenges for all these networks is a shift from a hospital-centric focus to a more prevention-based child health technology model, as the focus on children’s health care is moving toward health prevention, community-based healthcare, and self-care and management. Moreover, ensuring that child health technologies are developed for primary care ensures that a larger volume of children are supported without the need to refer into secondary care for specialist input.

## 3 Artificial intelligence in paediatric healthcare – opportunities and challenges

Artificial intelligence (AI) has the potential to transform paediatric healthcare, offering unprecedented capacity to interrogate complex, high-dimensional datasets, provide predictive short and long-term analytics and personalise healthcare for CYP. Contemporary AI systems can integrate multimodal inputs, including medical imaging, physiological parameters, genomic data, and unstructured free-text clinical from clinical notes to generate clinically actionable insights. These applications span diagnostic support, risk stratification, treatment optimisation, and workflow automation, thereby enhancing both precision and efficiency within paediatric care pathways (23–25). Unlike adult medicine, paediatrics requires continuous adaptation to dynamic physiological and developmental changes, which complicates conventional clinical decision-making. AI systems, through advanced ML and deep learning methodologies, can model these non-linear trajectories and provide personalised predictions that support clinicians in tailoring interventions to the unique needs of CYP (26–28).

### 3.1 AI-enabled diagnostics

Artificial intelligence has demonstrated high accuracy in detecting paediatric conditions across multiple settings. In medical imaging, deep convolutional neural networks have achieved performance metrics comparable to, and in some cases exceeding, expert radiologists in the identification of conditions such as pneumonia (29), brain tumours (30), retinopathy of prematurity (31), cardiac conditions (32) and asthma (33). Moreover, reducing scan time, lowering radiation dose, and improving image quality (by minimising noise and motion or metal artefacts) have been key areas of research in MRI and CT technology, by using AI-supported deep-learning tools to reduce scanning times (34) and the need for intravenous contrast (35), all of which benefit children. Importantly, shorter MRI acquisition times would not only increase daily throughput by enabling a greater number of studies but also diminish motion artefacts in less co-operative paediatric patients and thereby reduce reliance on general anaesthesia, mitigating its associated risks.

Another example is AI-driven electroencephalographic (EEG) analysis has facilitated the early detection of neonatal seizures and enabled predictive modelling of long-term neurodevelopmental trajectories (36). By applying machine learning algorithms to temporal and spectral EEG features, these systems provide clinicians with probabilistic risk assessments that support timely intervention and prognostic counselling. In the field of neurodevelopmental paediatrics, ML approaches integrating multimodal behavioural data, speech patterns, and eye-tracking metrics have shown considerable promise in the early identification of autism spectrum disorder (ASD) (37). Such models operationalise complex behavioural phenotypes into quantifiable features, enabling earlier recognition of atypical developmental trajectories than is typically achievable through standard clinical assessment. Collectively, these AI-enabled diagnostic modalities have the potential to mitigate diagnostic latency, particularly in resource-constrained or underserved regions where paediatric subspecialist expertise is limited, by providing scalable, reproducible, and data-driven diagnostic support.

### 3.2 AI-driven predictive analytics and precision paediatrics

Predictive analytics and precision paediatrics represent two converging domains within child health technology, each leveraging AI to transform clinical decision-making, risk stratification, and personalised care. AI-driven predictive models aim to synthesise complex, multimodal datasets, that includes data from physiological monitoring, environmental sources, and electronic health records to identify CYP who may be at risk of clinical deterioration or complications, hospital readmission, or adverse drug reactions. For example, in paediatric intensive care units, early warning systems trained on continuous monitoring streams can detect sepsis several hours before overt clinical manifestation, supporting proactive escalation, although at present there is limited evidence that these systems will ultimately improve overall survival rates (38, 39). Similarly, the pCART machine-learning–based Paediatric Early Warning Score can predict which children on the ward are at high risk of deterioration and requiring ICU-level care within 12 hours. By analysing vital signs and clinical data to predict deterioration earlier than standard methods, the model enabled faster intervention and reduced the rate of serious events such as invasive ventilation, need for vasoactive support, or death and reduced critical events by more than two-thirds.

Data from environmental and climatic factors is also been trialled to predict symptom escalation in long-term conditions. Several studies have sought to leverage environmental data as well as physiological parameters including lung function trends, symptom scores, and medication use patterns to predict the likelihood of future exacerbations in patients with asthma, with some ML models demonstrating utility in exacerbation prediction (40, 41). Similarly, in paediatric allergy and immunology, ML models that integrate environmental data have demonstrated significant potential in forecasting allergen exposure and supporting the prevention of acute allergic reactions and wheeze (42).

Parallel advances in precision paediatrics further illustrate the transformative potential of AI. Multi-omic integration, advanced imaging, and detailed phenotypic data allow AI systems to accelerate the diagnosis of rare diseases. Facial phenotyping tools such as Face2Gene, support the recognition of genetic syndromes that have the potential to improve diagnostic accuracy and reduce diagnostic delay (43). Face2Gene is commercially available and can identify the correct genetic condition within ten suggested conditions 95% of the time over a total of more than 200 genetic diagnoses. In paediatric neuro-oncology, radiomics has emerged as a powerful tool for extracting quantitative imaging features that support tumour histology determination, identification of disseminated disease, prognostication, and molecular classification through radiogenomic approaches. AI-enabled radiomics further enhances these capabilities by integrating imaging-derived signatures with genomic and clinical data to improve tumour classification, guide treatment planning, and predict therapeutic response. However, despite its promise, the shift of precision paediatrics into clinical care has significant methodological challenges, including small and heterogeneous paediatric sample sizes, limited generalisability of predictive models, and the need for robust data harmonisation across imaging centres, highlighting the need for rigorous, standardised approaches to ensure reliable clinical support (44).

Other areas where AI may improve diagnostic capabilities is in the field of microRNAs (miRNAs). MiRNAs are short, non-coding RNA molecules that regulate gene expression post-transcriptionally, acting as key modulators of developmental, physiological, and disease processes. MiRNA expression patterns can reflect underlying developmental, pathological, and treatment-related processes, offering new opportunities for early diagnosis, risk stratification, and personalised therapeutic monitoring in children. By integrating miRNA profiling with broader multi-omic and clinical datasets, this field is moving toward more accurate, biologically informed models of paediatric disease, although challenges remain in standardisation, validation, and translation to routine clinical practice (45).

Collectively, these and other AI/ML developments in paediatrics demonstrate how predictive analytics and precision paediatrics, underpinned by robust methodologies, can shift paediatric healthcare from reactive, episodic intervention toward anticipatory, personalised, and data-driven models of care (46, 47).

### 3.3 Challenges, bias, safeguarding, and rights-based considerations in paediatric AI

The integration of AI and ML into paediatric healthcare presents transformative opportunities, but it also introduces a series of methodological, ethical, and rights-based challenges (Table 2). A central concern is algorithmic bias, which arises when training datasets under-represent key paediatric subgroups including neonates and infants, minority ethnic groups, and CYP with disabilities, leading to systematically biased predictions and inequitable outcomes. Paediatric datasets are often smaller, more fragmented, and less representative than adult datasets, increasing the risk that AI systems may inadvertently reinforce existing structural disparities in child health services (17). A second major challenge is model explainability within in AI/ML algorithms; clinicians, CYP, and families are now calling for transparent and interpretable models to support safe and trustworthy decision-making. Black-box algorithms undermine clinical confidence, complicate shared decision-making, and hinder regulatory approval (48, 49). This is particularly salient in paediatrics, whereby decisions often involve parents or guardians and where developmental considerations shape risk–benefit assessments. To counter this, explainable AI (XAI) methodologies are now being used including SHAP, LIME, Grad-CAM, and attention maps (50) to ensure that developers and users understand how an AI algorithm has arrived at its decision. Equally important is the need for rigorous, age-specific validation. Paediatric AI systems must be evaluated across developmental stages, diverse clinical settings, and heterogeneous populations to ensure generalisability and safety ensuring that AI systems address the biological, psychological and developmental progression seen during childhood (51, 52).

**Table 2:** Summary of the key methodological, ethical, and rights-based challenges associated with the development and deployment of AI systems in paediatric healthcare.

Domain	Issue	Description	Implications for practice
Algorithmic bias	Under-representation of key paediatric subgroups	Paediatric datasets often exclude neonates, minority ethnic groups, CYP with disabilities, and rare conditions.	Leads to inequitable predictions and reinforces structural disparities.
Data quality	Small, fragmented, heterogeneous datasets	Variability across EHRs, wearables, imaging, and behavioural data reduces model robustness.	Increases error rates and limits generalisability.
Explainability	Black-box models	Lack of transparency undermines clinician trust and shared decision-making with families.	Requires XAI tools (SHAP, LIME, Grad-CAM) to support safe clinical use.
Age-specific validation	Developmental variability	Physiological and psychological changes across childhood require stage-specific evaluation.	Models must be validated across age bands and diverse settings.
Safeguarding	Continuous monitoring and digital phenotyping	Wearables and remote sensing may create a sense of surveillance and psychological burden.	Requires clear boundaries, escalation protocols, and CYP-appropriate consent.
Privacy	Longitudinal data and re-consent challenges	CYP cannot provide long-term consent; re-contacting them later is often impossible.	Necessitates privacy-preserving approaches and minimal-data design.
Autonomy and agency	Behavioural nudges and persuasive design	AI-driven prompts may influence CYP in ways they cannot fully understand or contest.	Requires rights-based design and transparency about automated influences.
Equity	Digital divide and structural inequalities	Families with limited connectivity, devices, or digital literacy face barriers to participation.	Tools must be designed for low-resource settings and evaluated for differential impact.
Cultural and linguistic adaptation	One-size-fits-all models	AI systems may not reflect cultural norms, languages, or communication styles relevant to CYP.	Requires localisation, co-design, and inclusive dataset development.
Regulatory oversight	Limited paediatric-specific guidance	Current AI regulations are adult-focused and overlook developmental needs.	Calls for paediatric-specific standards and lifecycle monitoring.
Psychological safety	Impact of monitoring and risk prediction	Crisis-risk algorithms may cause anxiety or stigma if poorly communicated.	Requires sensitive communication and clinician-mediated interpretation.
Clinical workflow integration	Misalignment with real-world practice	Tools that add burden or disrupt workflow are rarely adopted.	Requires co-design with clinicians and implementation-science approaches.
Human–AI interaction	Over-reliance or under-reliance on AI	Clinicians may over-trust or ignore AI outputs depending on design and training.	Requires training, calibration, and clear role definitions.
Liability and accountability	Unclear responsibility for AI-driven decisions	Ambiguity over whether clinicians, developers, or organisations are accountable for errors.	Requires explicit governance, documentation, and medico-legal clarity.
Emotional and developmental impact	CYP understanding of AI-mediated care	Children may misinterpret automated decisions or monitoring as punitive or threatening.	Requires developmentally appropriate communication and supportive framing.
Interoperability	Fragmented systems	AI tools may not integrate with existing EHRs or community systems.	Limits scalability and increases clinician burden.
Data drift and model decay	Changing paediatric populations	Growth, development, and evolving disease patterns can cause model performance to degrade.	Requires continuous monitoring, recalibration, and post-market surveillance.

Beyond these technical considerations, AI in paediatrics raises unique rights-based and safeguarding concerns. Privacy is a major issue, as CYP have limited capacity to provide informed consent for long-term data use, yet AI systems often rely on large-scale, longitudinal datasets that may follow individuals across childhood and adolescence. Moreover, reconsenting children when they are older is particularly challenging as they may not be contactable at a later date. Autonomy and developmental agency may also be affected, as AI-driven nudges or behavioural interventions can influence CYP in ways they may not fully understand or be able to contest. Surveillance risks also arise from continuous monitoring technologies such as wearables, digital phenotyping tools, and remote sensing systems which may create a persistent sense of being observed, with potential impacts on psychological wellbeing (Table 2).

Given the rapid growth in digital health, there is a growing risk of widening existing child health inequalities if structural, social, and economic factors are not explicitly addressed during technology development and evaluation. Digital tools, while often promoted as universally beneficial, can disproportionately advantage families with stable connectivity, adequate devices, digital literacy, and supportive home environments, while leaving behind those facing poverty, disability, language barriers, or unstable housing. Children’s digital participation is shaped not only by access to technology but also by the broader ecosystem of support around them, including parents’ confidence, school resources, and community infrastructure (17).

Collectively, these challenges underscore the need for robust governance frameworks, such as ACCEPT-AI, that embed equity, transparency, developmental appropriateness, and rights-based protections into every stage of paediatric AI development (53). Alongside technical standards and proposed governance for dataset quality, validation, and explainability, there is a parallel call for minimum conditions for digital participation in childhood, articulated through the emerging concept of a ‘minimum digital living standard’ which specifies the infrastructure, skills, and support families need for children to benefit safely and meaningfully from digital health solutions (54). Therefore, ensuring that AI systems are safe, fair, and trustworthy for children and young people requires not only technical excellence but also ethical design, inclusive data practices, attention to digital capability and access, and meaningful engagement with children, families, and multidisciplinary clinical teams throughout the AI lifecycle.

## 4 Digital therapeutics and immersive technologies

Digital therapeutics (DTx) and immersive technologies constitute a rapidly evolving field in paediatric healthcare innovation (55, 56). These interventions deliver evidence-based therapeutic content through software platforms that integrate behavioural science, gamification, adaptive algorithms, and real-time feedback. In paediatrics, DTx and immersive modalities are increasingly deployed to support mental health, neurodevelopmental conditions, chronic disease management, procedural care, and rehabilitation (17, 57–64). Their scalability and capacity for personalisation position them as important adjuncts to conventional clinical pathways in children’s healthcare, particularly in contexts of rising demand and constrained specialist capacity, albeit larger scale randomised control studies are required to demonstrate their effectiveness compared to conventional therapies before the introduction into real-world clinical settings (65–67).

### 4.1 Software-based therapeutics

Software-based DTx products are gaining traction across multiple paediatric conditions including mental and physical health. For example, in attention-deficit/hyperactivity disorder (ADHD), FDA-cleared digital cognitive training tools have demonstrated improvements in attention and executive functioning, offering a non-pharmacological adjunct for children with attention dysregulation (68). App-based cognitive behavioural therapy (CBT), mood-tracking platforms, and guided self-help interventions are also increasingly being used to support children and adolescents with anxiety and depression, providing structured, accessible psychological support outside traditional clinic settings (69–71). Digital pain-management programmes incorporating biofeedback and behavioural strategies have shown benefit in chronic pain conditions (58, 72, 73), while digital coaching and personalised behavioural nudges are being used to support glycaemic control in paediatric diabetes (74–76). Digital mental health tools are now increasingly deployed to address rising rates of anxiety, depression, and self-harm observed in children and young people. These include AI-supported triage systems that use digital phenotyping to identify individuals at risk of crisis, chat-based interventions that provide guided psychoeducation and emotional support, and gamified CBT platforms designed to enhance engagement and adherence (70, 77, 78). While these tools offer potential for early intervention and scalable support, their clinical deployment requires rigorous evaluation to ensure safety, developmental appropriateness, and cultural relevance.

### 4.2 Immersive therapeutics (VR/AR)

Immersive technologies, including virtual reality (VR) and augmented reality (AR) have demonstrated therapeutic efficacy across a broad range of paediatric applications. Systematic reviews show that VR significantly reduces procedural pain and anxiety during blood draws, wound care, and other distressing interventions, with several analyses confirming reductions in analgesic requirements and improved procedural compliance (79–81). AR has similarly been shown to enhance paediatric patient outcomes by improving engagement, reducing distress, and supporting procedural cooperation in clinical environments (82). VR-based rehabilitation programmes have been used to support upper and lower limb rehabilitation by recapitulating movements from conventional physiotherapy into immersive gamification to promote adherence and reduce pain (63, 64); and in the field of children’s mental health, immersive graded-exposure environments have also been applied to phobia treatment, enabling controlled, developmentally appropriate exposure to feared stimuli (83, 84). Beyond clinical therapy, VR and AR are now increasingly being developed for education and self-management, providing interactive simulations that enhance disease understanding and coping skills (85–87).

### 4.3 Challenges: evidence, regulation, and long-term impact

Despite the rapid growth in this field, several challenges constrain the safe and effective implementation of DTx and immersive technologies in paediatrics. Evidence generation remains limited, with many products lacking long-term, high-quality clinical trials in paediatric populations. Regulatory frameworks for software as a medical device (SaMD) vary across jurisdictions and often lack paediatric-specific guidance, creating uncertainty for developers and clinicians. Sustained engagement is also a persistent challenge, particularly among adolescents, where novelty effects may diminish over time.

Importantly, poorly designed, or unregulated technologies have the potential to cause harm by exacerbating anxiety, reducing help-seeking, or providing inaccurate or developmentally inappropriate advice (88). These risks underscore the need for rigorous evaluation, transparent reporting, and ongoing post-market surveillance and underpins importance of ethical and rights-based considerations in DTx and AR/VR development by which CYP and their parents should have meaningful input into the design, evaluation, and use of digital interventions, ensuring that tools align with their developmental needs and preferences. Sensitive mental health and behavioural data require stringent privacy safeguards, particularly given the long-term implications of digital footprints created in childhood. DTx must also be accessible to diverse populations, including those with disabilities, limited digital access, or socioeconomic disadvantage and, finally, digital tools must augment the therapeutic relationships that underpin safe and effective paediatric care (89, 90). A CYP rights-based approach ensures that DTx promote wellbeing without compromising dignity, privacy, or equity.

## 5 Wearables and biosensing in paediatrics

Wearable technologies and biosensing systems can enable continuous, non-invasive monitoring of physiological, behavioural, and environmental parameters. These devices generate high-resolution data streams that support early detection, personalised care, and proactive intervention that are potentially amenable to algorithmic processing by AI/ML (91, 92). CYP with long-term conditions, and those requiring long-term monitoring stand to benefit particularly from these innovations, which shift care from episodic, clinic-based encounters toward continuous, child-centred models of support, and a potential shift of their healthcare from the hospital to the community (92). Physiological monitoring is a core tenet of paediatric wearables. Modern biosensors can capture heart rate, respiratory rate, oxygen saturation, temperature, electrodermal activity, and movement patterns, supported by advances in flexible electronics, textile-embedded sensors, and skin-adhesive devices that enhance comfort and durability for paediatric use. These technologies are increasingly deployed for respiratory monitoring in asthma, bronchiolitis, and sleep-disordered breathing; cardiac monitoring in arrhythmias, congenital heart disease, and post-operative recovery; and neurological monitoring for seizure detection, developmental assessment, and behavioural phenotyping (91–93). Collectively, these systems reduce reliance on hospital-based monitoring, support earlier discharge, and enable safe home-based care.

In paediatrics digital biomarkers produced by wearable sensors are being used in the early detection and management of long-term conditions – in improving asthma control by using respiratory patterns, cough frequency, and environmental exposures; for epilepsy using accelerometry and electrodermal activity to detect seizures (93); for autism spectrum disorder using movement signatures identifying automated and stereotypical behaviours (94–96), eye tracking (97), and vocal features (98); and for mental health using sleep regularity (99, 100), activity rhythms, and social interaction patterns as indicators of wellbeing (100, 101). These biomarkers provide objective, continuous measures that complement traditional clinical assessments, which are often episodic, subjective, and influenced by recall bias.

Taking this one step further, remote monitoring systems can integrate wearable sensors with cloud-based analytics, AI-driven trend detection, and clinician-facing dashboards. These platforms underpin emerging models of care such as virtual paediatric wards, enabling early discharge and home-based monitoring for acute- and long- term conditions (102). They also support early detection of deterioration through AI-supported alerts and promote family-centred care by empowering parents and caregivers to participate actively in monitoring and decision-making (92), aligning with broader shifts toward proactive, preventative, and digitally enabled care.

Despite their promise, paediatric wearables face several unique challenges. Physiological parameters vary substantially across developmental stages, necessitating age-specific calibration and validation to ensure accuracy. Devices must be comfortable, durable, and acceptable to children with sensory sensitivities or neurodevelopmental differences, while data quality may be compromised by motion artefacts, inconsistent wear time, and environmental noise (91). Equity remains a critical concern, as variability in access to devices, connectivity, and digital literacy risks exacerbating existing health disparities (103). Addressing these limitations will require rigorous paediatric-specific validation, inclusive design processes, and governance frameworks that ensure safety, equity, and developmental appropriateness. As wearable technologies become increasingly integrated with AI-enabled analytics, the importance of transparent algorithms, robust data stewardship, and rights-based safeguards will be amplified. Ensuring that these systems enhance children’s autonomy, privacy, and wellbeing is also essential. When developed and deployed responsibly, wearables and biosensing systems have the potential to transform paediatric healthcare by enabling earlier intervention, supporting personalised management, shifting care to community- and home- based settings and strengthening the partnership between families and clinical teams.

## 6 The evolving paradigm of precision paediatrics

Precision paediatrics represents a paradigm shift in how we view children’s healthcare, moving beyond traditional population-level approaches in the management of health and disease, toward interventions that are tailored to the unique biological, developmental, environmental, and social contexts of CYP. Unlike adult precision medicine, which is often driven by genomic stratification and targeted therapeutics, precision paediatrics must account for the dynamic physiology of growth, maturation, and neurodevelopment, as well as the profound influence of family, education, and social determinants on health trajectories (2). This requires analytical frameworks and digital infrastructures capable of integrating multi-omic data, longitudinal phenotyping, real-world evidence, and lived experience to generate actionable insights that are developmentally appropriate and clinically meaningful (104–106). Given the complexity of this process, quantum and federated AI may be required to achieve this, by markedly improving processing speed and allowing AI access to data to support analysis without the need to move datasets into cloud-based systems. Quantum architectures offer the potential to handle the combinatorial scale of multi-omic, imaging, and continuous sensor streams, enabling simulations and predictive models that would be computationally prohibitive using classical methods. Federated AI, meanwhile, allows these analyses to occur across distributed clinical, community, and home-monitoring environments while keeping sensitive paediatric data local, thereby reducing privacy risks and supporting compliance with stringent data-protection requirements.

At the molecular level, advances in genomics, epigenomics, proteomics, metabolomics, and emerging fields such as miRNA profiling (see earlier) are enabling earlier detection of disease risk, more accurate diagnostic classification, and refined prognostication (107, 108). These approaches are particularly powerful in paediatric oncology (109–112), rare disease diagnostics (113, 114), and immune-mediated conditions (115), whereby early identification of pathogenic pathways can alter lifelong outcomes. However, the interpretation of molecular signatures in children requires age-specific reference ranges, developmental calibration, and careful consideration of evolving physiology. Precision paediatrics therefore demands analytical models that explicitly incorporate age, growth, and physical and developmental maturation as biological variables rather than confounders. Large datasets will be required to achieve this goal.

Digital technologies, including wearable sensors, remote monitoring platforms, and AI-enabled clinical decision support, are accelerating the transition toward proactive, personalised care (2). Continuous physiological monitoring, digital biomarkers, and adaptive algorithms can detect early deviations from expected developmental or disease trajectories, enabling timely intervention and reducing the burden of acute deterioration, alongside the gathering of information from the environment, a process now termed exposomics (116–119). In long-term conditions such as diabetes, asthma, epilepsy, and neurodevelopmental disorders, precision digital tools have the potential to support individualised treatment plans, behavioural coaching, and real-time feedback that align with each child’s developmental stage and daily environment (120).

Importantly, precision paediatrics also extends beyond physical health to encompass the psychosocial and structural determinants that shape child health. Integrating environmental exposures, social context, educational engagement, and family-level factors into predictive models allows for more holistic risk stratification and targeted support. Precision approaches must therefore be inclusive, culturally responsive, and attentive to digital inequities (121). Ultimately, precision paediatrics will not be defined by technology alone but by its commitment to delivering the right intervention, at the right time, in the right way, for every child (2). Achieving this vision requires robust governance, interdisciplinary collaboration, and sustained investment in paediatric-specific research, data infrastructure, and workforce capability (121). When implemented responsibly, precision paediatrics has the potential to transform child health, reduce lifelong morbidity, improve future health and prosperity, and create a more equitable and responsive healthcare system for future generations.

## 7 Regulatory science for paediatric digital health

Regulatory science is fundamental to ensuring that paediatric digital health technologies are safe, effective, and trustworthy. However, regulatory frameworks have struggled to keep pace with the rapid evolution of AI, biosensing, digital therapeutics, and precision medicine (122, 123). These challenges are amplified in paediatrics, where developmental and anatomical variability, ethical constraints, and heightened safeguarding requirements demand more stringent oversight (124, 125). As digital tools increasingly support and influence diagnosis, monitoring, and treatment, regulators must adapt current regulatory frameworks to address the unique risks and opportunities presented by child-focused technologies in the same way that the paediatric regulations in the pharmaceutical industry have evolved (125, 126). AI-enabled tools and digital therapeutics are typically governed under software-as-a-medical-device (SaMD) frameworks. Major regulatory bodies including the FDA, EMA, and MHRA have issued guidance on AI/ML-based SaMD, but frameworks tailored to paediatrics are still largely undeveloped (127–130). Several challenges complicate regulatory evaluation. Dynamic, self-modifying algorithms exceed the capacity of the current AI/ML regulatory pathways, necessitating more flexible oversight structures, because their behaviour can evolve after deployment in ways that cannot be fully characterised at the point of approval. As these AI/ML systems continue to learn from new data, their performance profile, risk characteristics, and decision-making boundaries may shift over time, meaning that traditional one-off pre-market assessments are insufficient to guarantee ongoing safety and effectiveness. This creates a regulatory imperative for lifecycle-based oversight, incorporating mechanisms such as continuous performance monitoring, and risk-stratified post-market evaluation to ensure that adaptive models remain clinically reliable as they evolve (127). Transparency and explainability in AI are increasingly mandated, particularly for high-risk clinical applications where previously opaque models have undermined clinician trust or patient safety, as regulators now recognise that clinicians must be able to understand how an algorithm reaches its outputs in order to validate its reliability, identify potential sources of error, and make safe, defensible decisions at the point of care (131). Furthermore, post-market surveillance is essential to detect performance drift, especially in dynamic paediatric populations whose physiology and behaviour change rapidly over time, as even small shifts in growth, developmental stage, or disease phenotype can alter how an algorithm interprets clinical data. Continuous or regular post-marketing monitoring of AI/ML therefore becomes critical to ensure that model outputs remain accurate, safe, and clinically appropriate as the underlying population evolves and develops. This reinforces the need for ongoing real-world performance evaluation and age-stratified monitoring to identify emerging risks early and maintain reliable decision-support across childhood (132–134). Regulators are therefore exploring novel approaches such as predetermined change-control plans, real-world performance monitoring, and risk-based adaptive oversight to ensure ongoing safety (127, 135). Because software updates can alter therapeutic content, regulators must therefore consider mechanisms for ongoing oversight beyond initial approval. Initiatives such as the FDA’s Digital Health Center of Excellence (136) and the EMA’s Innovation Task Force (137) are developing frameworks to support safe, evidence-based deployment of DTx in children.

Finally, paediatric digital health is inherently global, with technologies frequently developed in one jurisdiction and deployed in another. Cross-border harmonisation is therefore essential to reduce duplication, accelerate innovation, and ensure consistent safety standards. Key initiatives include the International Medical Device Regulators Forum (IMDRF) guidance on SaMD (127), the Global Digital Health Certification Network (GDHCN) (138), and the proposed EU–US collaborations on AI regulation and data governance. Harmonised approaches will be critical to supporting safe, equitable, and scalable digital health innovation for children and young people.

## 8 Ethical and rights-based considerations

Ethical and rights-based considerations are central to the development, deployment, and governance of paediatric digital health technologies. Children are a uniquely vulnerable population with evolving capacity, capability, and autonomy, and who are susceptible to privacy and surveillance risks. The United Nations Convention on the Rights of the Child (UNCRC) provides a foundational framework for the design and development of ethical digital health innovation, emphasising the rights to protection, participation, privacy, and equitable access (139). As digital tools become increasingly embedded in paediatric care, these principles must guide design, evaluation, and implementation to ensure that technologies enhance children’s health, healthcare, and wellbeing, whilst respecting and supporting their dignity, and agency (139, 140).

### 8.1 Managing data

Whilst data will provide the foundation for managing the current and future health and wellbeing of CYP, the ramifications of storing data must be considered in the context of its future use and impact on the background of an evolving digital world becoming increasingly more influenced by AI. Data collected early in life may influence future opportunities in education, employment, insurance, and social participation. Informed consent is particularly complex as CYP will not fully understand the implications of data sharing, while parental consent may not always reflect the child’s preferences or best interests, and this raises important future considerations as the child matures. Decisions made on their behalf today may create long-term digital footprints, influence future autonomy, and shape how their health data can be used, linked, or reinterpreted over time. This underscores the need for developmentally appropriate consent models, mechanisms for children to assume control of their data as they age, and ongoing review of consent validity to ensure that early decisions remain ethically and legally defensible throughout adolescence and adulthood (53, 141). In addition, currently established privacy-preserving machine learning techniques such as federated learning, differential privacy, and secure multi-party computation offer important safeguards for paediatric digital health (142, 143). Federated learning enhances security by keeping raw data within local clinical or home environments, allowing models to be trained without centralising sensitive paediatric records, thereby reducing the risk of large-scale data breaches, and limiting unnecessary exposure of identifiable information. By enabling collaborative model development while maintaining strict data locality, federated approaches also support future-facing requirements for privacy-preserving AI and distributed governance models, which are likely to become increasingly important as paediatric datasets grow, diversify, and integrate across health, education, and home-monitoring ecosystems (142–144). Differential privacy provides mathematical protection against re-identification by adding carefully calibrated noise to data or model outputs, ensuring that no individual child can be singled out, even when datasets are small or contain rare conditions. By limiting how much any single record can influence the final result, it offers a clear and measurable privacy guarantee. This makes it particularly valuable in paediatrics, where data are often highly sensitive and children may be more easily identifiable (145, 146). In addition, secure multi-party computation enables multiple institutions to collaborate on shared analyses without exposing their underlying datasets, preventing any single party from accessing complete identifiable information, because the data remain encrypted throughout the process and are never revealed in raw form. Each organisation contributes only the encrypted components needed for the joint calculation, meaning the final result can be generated without compromising the privacy of individual children. This makes it a valuable approach for cross-site paediatric research, where data are often sensitive and distributed across many settings (147, 148). Together, these approaches reduce the risk of data breaches, limit exposure of highly sensitive physiological and behavioural data, and aim to support ethically robust AI development in paediatric healthcare.

### 8.2 Algorithmic fairness and digital autonomy

Algorithmic fairness is another critical ethical concern (149). AI systems risk perpetuating or amplifying health inequities particularly when training datasets under-represent paediatric subgroups (53, 150, 151). Ethical AI in paediatrics therefore requires diverse and representative datasets, systematic bias detection and mitigation strategies, transparent reporting of model performance across demographic and clinical subgroups, and ongoing monitoring for performance drift as populations and contexts evolve (53). One practical way to mitigate this algorithmic bias is to implement stratified data augmentation and targeted data collection, deliberately oversampling under-represented subgroups and integrating synthetic but biologically plausible data where appropriate (152, 153). This can be combined with subgroup-specific model calibration, ensuring that algorithms are validated separately for specific age-groups including neonates, infants, adolescents, minority ethnic groups, CYP with disabilities, and other marginalised populations (154). Together, these approaches help ensure that model performance is equitable across diverse paediatric groups and reduce the likelihood that AI systems will systematically disadvantage those already at risk of poorer health outcomes.

Digital health tools must also respect the evolving capacities of CYP. Younger children require parental involvement in decision-making, yet their preferences should still be considered (53, 139, 155). Similarly, neurodivergent children may have unique sensory, communication, or privacy requirements that necessitate tailored design approaches. Adolescents, by contrast, place high value on autonomy and may resist tools perceived as controlling, intrusive, or misaligned with their developmental needs (155). Therefore, ethical digital health design must balance protection with empowerment, ensuring that technologies support agency, independence, and self-efficacy. A practical way to mitigate these risks is to adopt developmentally attuned, choice-rich design strategies that adapt to the evolving capacities of CYP. This includes offering graduated levels of autonomy, where younger children engage through simplified interfaces and shared decision-making with parents, while adolescents can access more independent modes with adjustable privacy settings and control over notifications, data visibility, and engagement frequency, encompassed in the process of dynamic consent (156). Dynamic consent incorporates personalised communication platforms designed to enhance participant engagement across clinical care and research. It is a participant-centred approach that positions patients and research contributors at the heart of decision-making, offering an interactive digital interface that supports meaningful, ongoing involvement. Neurodivergent CYP may benefit from sensory-adaptive interfaces, multimodal communication options, and customisable interaction pathways that respect diverse cognitive and sensory profiles. Embedding dynamic consent and assent mechanisms allows CYP to revisit and modify their preferences over time, further supporting their agency and overcoming the previously described issues of static, one-off consent models that fail to account for children’s evolving understanding, values, and autonomy. By enabling preferences to be updated as circumstances change, these approaches help ensure that data-sharing decisions remain aligned with the child’s best interests throughout development.

Equity and digital inclusion are additional ethical imperatives. Access to devices, connectivity, digital literacy support, culturally relevant content, and accessible interfaces varies widely across socioeconomic, geographic, and demographic groups. Without intentional design and targeted interventions, digital transformation risks widening existing health disparities. Ethical digital health therefore requires proactive strategies to ensure that all children, irrespective of their background and challenges, can benefit from digital innovation, including those with disabilities, those living in deprived or remote areas, and those from minoritised communities who may face additional structural barriers. Embedding equity from the outset helps ensure that digital tools do not simply replicate existing inequalities but actively contribute to reducing them (54).

### 8.3 Safeguarding considerations and maintaining psychological well-being

Safeguarding and psychological wellbeing are central to ethical digital health practice. Digital tools can provide reassurance, continuity, and timely support, but they may also introduce new risks. Remote monitoring may reduce anxiety for some families while increasing stress for others. For example, digital mental health tools may offer valuable support but risk misinterpretation, delay help-seeking, or expose children and young people to content that is not developmentally appropriate, particularly when used without adequate guidance or when alerts and feedback are difficult for families to interpret. These mixed effects highlight that digital tools can both reassure and overwhelm, depending on the child’s developmental stage, the family’s digital literacy, and the level of clinical oversight available (157, 158). Furthermore, AI-driven behavioural nudges may influence choices in ways that are insufficiently transparent or ambiguous, shaping how children, young people, or their families respond to prompts without fully understanding the underlying logic or intent. Such nudges can subtly steer decision-making, potentially reinforcing biases or encouraging actions that do not align with the child’s preferences or best interests.

Mitigating these risks therefore requires safeguarding frameworks that are proactive, developmentally aligned, and embedded throughout the digital health ecosystem (53). Clear escalation pathways must ensure that concerning symptoms, negative behavioural changes, or safety signals detected by digital tools trigger timely clinical review rather than depending on automated responses alone. Human oversight (known as human in the loop in digital/AI terms) is essential, with trained professionals available to interpret data, contextualise alerts, and intervene when digital tools identify potential risk (23, 25). Age-appropriate content standards should be rigorously applied, ensuring that information, prompts, and behavioural nudges are developmentally suitable, culturally sensitive, and free from coercive or opaque influence (139–141). Digital tools should incorporate mechanisms that allow CYP to express discomfort, report harm, or request clarification, alongside accessible options to pause, withdraw consent, or opt out entirely without penalty (159). Regular psychological safety assessments, co-designed with CYP, could also help identify unintended harms such as increased anxiety, over-monitoring, or reduced help-seeking. Taken together, these considerations highlight why it is essential that the development of digital regulatory standards for children explicitly incorporates principles of psychological safety, developmental appropriateness, and meaningful participation. Embedding these protections within future frameworks ensures that digital health technologies do not simply meet technical benchmarks but also uphold children’s rights, safeguard wellbeing, and promote equitable access to safe, trustworthy innovation. In doing so, regulatory standards can move beyond risk mitigation alone and actively shape a digital ecosystem that supports healthy development and responsible innovation for all CYP.

## 9 Co-design and co-development with children and young people (CYP)

Co-design and co-development are fundamental components of responsible paediatric digital health innovation (160). The UNCRC emphasises the rights of CYP to participate in decisions that affect them, recognising their evolving capacities and the importance of ensuring that their views are heard, taken seriously, and meaningfully incorporated into processes that shape their lives. This principle applies equally to digital health, where CYP should have opportunities to express preferences, raise concerns, and influence how technologies are designed, deployed, and governed. Embedding child participation as a core requirement strengthens legitimacy, improves acceptability, and ensures that digital systems reflect the needs and experiences of those they are intended to support (160, 161). In practical terms, participatory methods including workshops, advisory panels, and lived-experience partnerships ensure that digital health tools reflect the real needs, preferences, and contexts of the CYP they are intended to serve. Embedding participation throughout the innovation lifecycle is essential for ethical, equitable, and effective paediatric technology development, assessment, and adoption, ensuring CYP and their families are not merely consulted at isolated points but are meaningfully involved from early problem-framing through to design, testing, evaluation, and implementation. Continuous engagement helps surface real-world needs, usability challenges, and unintended consequences that may not be apparent to adult designers or clinicians. Thus, by integrating co-design practices, participatory evaluation, and ongoing feedback mechanisms ensures that paediatric technologies evolve in ways that are responsive, rights-respecting, and aligned with the populations they aim to serve (160, 162, 163). Importantly, co-design offers substantial benefits for digital health innovation. It improves usability by ensuring that technologies are intuitive, engaging, and aligned with children’s cognitive and sensory needs, reducing friction in everyday use, and increasing the likelihood that tools will be adopted and sustained over time. Co-design also helps identify barriers that adults may overlook, such as confusing navigation, overwhelming information, or features that do not reflect children’s real-world contexts. By embedding child-centred design principles and iterative feedback loops, developers can create solutions that are not only technically robust but genuinely meaningful and accessible to the CYP who rely on them (164). Moreover, digital acceptability increases when CYP recognise their own perspectives in the tools they use, fostering trust and sustained engagement (160, 165), and co-design enhances accuracy by ensuring that digital biomarkers, behavioural metrics, and AI models reflect real-world behaviours and contexts (163) (Table 3).

**Table 3:** Summary of the key benefits of involving CYP meaningfully throughout the digital health innovation process, highlighting how co-design and co-development enhances usability, relevance, equity, trust, and real-world validity while upholding children’s rights to participation and influence.

Dimension	Value	Explanation
Rights-based participation	Upholds CYP’s rights	Ensures CYP’s views are heard, respected, and embedded throughout innovation, aligning with UNCRC Articles 12 and 13.
Relevance and acceptability	Produces meaningful, context-aligned tools	CYP surface real-world needs, preferences, and challenges that adults may overlook, improving acceptability and everyday usability.
Usability and engagement	Enhances intuitive, child-centred design	Co-design ensures tools match cognitive, sensory, and developmental needs, increasing sustained engagement and reducing friction.
Accuracy and real-world validity	Improves digital biomarkers and AI models	CYP help ensure behavioural metrics and digital signals reflect authentic contexts, reducing bias and improving model performance.
Equity and inclusion	Reduces disparities	Targeted outreach and accessible formats ensure seldom-heard CYP (e.g., those in care, disabled, minoritised groups) shape innovation.
Trust and transparency	Builds confidence in digital systems	Genuine partnership reduces fears of surveillance or misuse and fosters shared ownership between CYP, families, and clinicians.
Iterative improvement	Supports continuous refinement	Ongoing feedback from CYP identifies usability issues, unintended consequences, and opportunities for improvement across the lifecycle.
Sustained engagement	Strengthens long-term involvement	Providing accessible follow-up and demonstrating impact of CYP contributions encourages continued participation and trust.

### 9.1 Principles of co-design with CYP

Traditional top-down design approaches by which technologies are soley conceived, designed, and implemented by adults, experts, or institutions frequently fail to capture the lived experiences, preferences, and developmental needs of CYP, resulting in technologies that are poorly aligned with their values, contexts, and expectations. Co-design directly addresses this gap by ensuring that CYP are active partners in shaping digital health tools, rather than passive recipients, and emphasises children’s rights to participation, expression, and influence in decisions that affect them (160, 162, 165).

Effective co-design with CYP is grounded in several core principles. It requires respect for evolving capacities, with methods tailored to developmental stages and communication preferences. Participation must be inclusive, ensuring representation across ages, abilities, cultural backgrounds, and lived experiences. Co-design must also involve genuine power-sharing, enabling CYP to shape decisions rather than merely provide feedback. Engagement should be iterative, occurring throughout the innovation lifecycle from defining unmet needs and problem definition to prototyping, testing, and evaluation. Furthermore, participation must be voluntary, supported, and emotionally safe. Fair compensation is important in recognising the expertise and labour contributed by CYP, and their confidentiality must be protected, particularly when they share sensitive experiences or perspectives. Participation should never feel obligatory or tokenistic; instead, CYP should be provided with the information, time, and support needed to contribute meaningfully and comfortably. Ensuring psychological safety, appropriate safeguarding measures, and clear confidentiality protections helps create environments where children and young people can speak openly without fear of judgement, exposure, or negative consequences (166–170). To address the needs of CYP within co-design, ethical frameworks must balance participation with protection, ensuring that they are empowered without being exploited. These principles reflect UNCRC Articles 12 and 13, which affirm the right of children to be heard and to express their views freely, while also requiring that adults create the conditions for those views to be shared safely and without undue pressure. Upholding these rights means providing CYP with genuine opportunities to contribute, ensuring they understand the purpose and implications of their involvement, and putting in place safeguards that prevent their insights from being misused (161).

### 9.2 A methodological approach to innovative co-design with children and young people

A wide range of methods can be used to engage CYP meaningfully in co-design (162, 171). Creative workshops, storyboarding, and prototyping allow children to visualise and shape concepts, making abstract ideas more concrete and accessible. Digital diaries and participatory design games provide engaging and developmentally appropriate ways for CYP to articulate needs, preferences, and concerns, especially for those who may find traditional interviews or focus groups intimidating. These approaches help surface insights that might otherwise remain hidden, support authentic expression, and ensure that design decisions are grounded in the lived experiences of CYP (172, 173). Alternatively, youth advisory panels and lived-experience partnerships offer sustained involvement and deeper insight into the realities of living with long-term conditions or navigating digital health systems. These ongoing structures allow CYP to contribute not only to early design decisions but also to iterative refinement, evaluation, and implementation. Their continuity supports richer dialogue, builds trust, and enables developers and clinicians to understand how digital tools function in everyday life, including the challenges, workarounds, and emotional impacts (17, 18, 74, 75, 174–176). Further approaches to ensure inclusion and minimise inequity include targeted outreach and facilitated participation for CYP who are seldom heard, such as those in care, those with disabilities, or those from minoritized communities (75, 164, 176). Providing accessible formats, including easy-read materials, visual supports, translation services, and alternative communication methods, enables broader engagement (164). Embedding trusted intermediaries, such as youth workers, teachers, or community advocates, can also help to support psychological safety and improves participation rates from groups who may mistrust formal research settings. Moreover, by offering flexible ways to take part in co-design and co-development such as remote sessions, whereby CYP are not required to be physically present, or by delivering workshops in schools or community settings, this ensures that CYP with restricted time, mobility challenges, or limited access to digital devices and connectivity are still able to engage meaningfully in the co-design process (176–178).

### 9.3 Co-design feedback

Providing structured follow-up after participation is critical for sustaining meaningful engagement (177). CYP consistently report that one of the most frustrating aspects of involvement in their healthcare or research is never hearing what happened next (179). Offering clear, timely updates about how their ideas were used, what decisions were made, and why certain suggestions were adopted or adapted demonstrates respect and accountability. This practice not only validates their contribution but also strengthens their confidence in digital health development and encourages future participation (180, 181). Importantly, feedback should be delivered in accessible, age-appropriate formats such as visual summaries, short videos, or interactive prototypes to ensure that CYP can understand and appreciate the impact of their involvement. Providing tailored feedback to minoritized groups is particularly important for ensuring that co-design processes are equitable and genuinely inclusive. Offering clear, culturally sensitive, and accessible feedback demonstrates respect for their contributions and helps build trust in systems that have not always served them equitably (182). By embedding feedback as a standard component of all technology co-design, developers and healthcare professionals can reinforce a culture of transparency, reciprocity, and genuine partnership with CYP.

Overall, co-design is also a powerful mechanism for building trust in digital health systems. By demonstrating respect for CYP’s perspectives, increasing transparency, and aligning technologies with lived experience, co-design reduces fears of surveillance, misuse, or misalignment with children’s needs. It strengthens relationships between CYP, families, clinicians, and developers, fostering a sense of shared ownership and mutual accountability. Trusted systems are more likely to be used, understood, and valued, enhancing both engagement and clinical impact.

## 10 The practical implementation of digital technologies in paediatrics and child health

**Figure 1:**
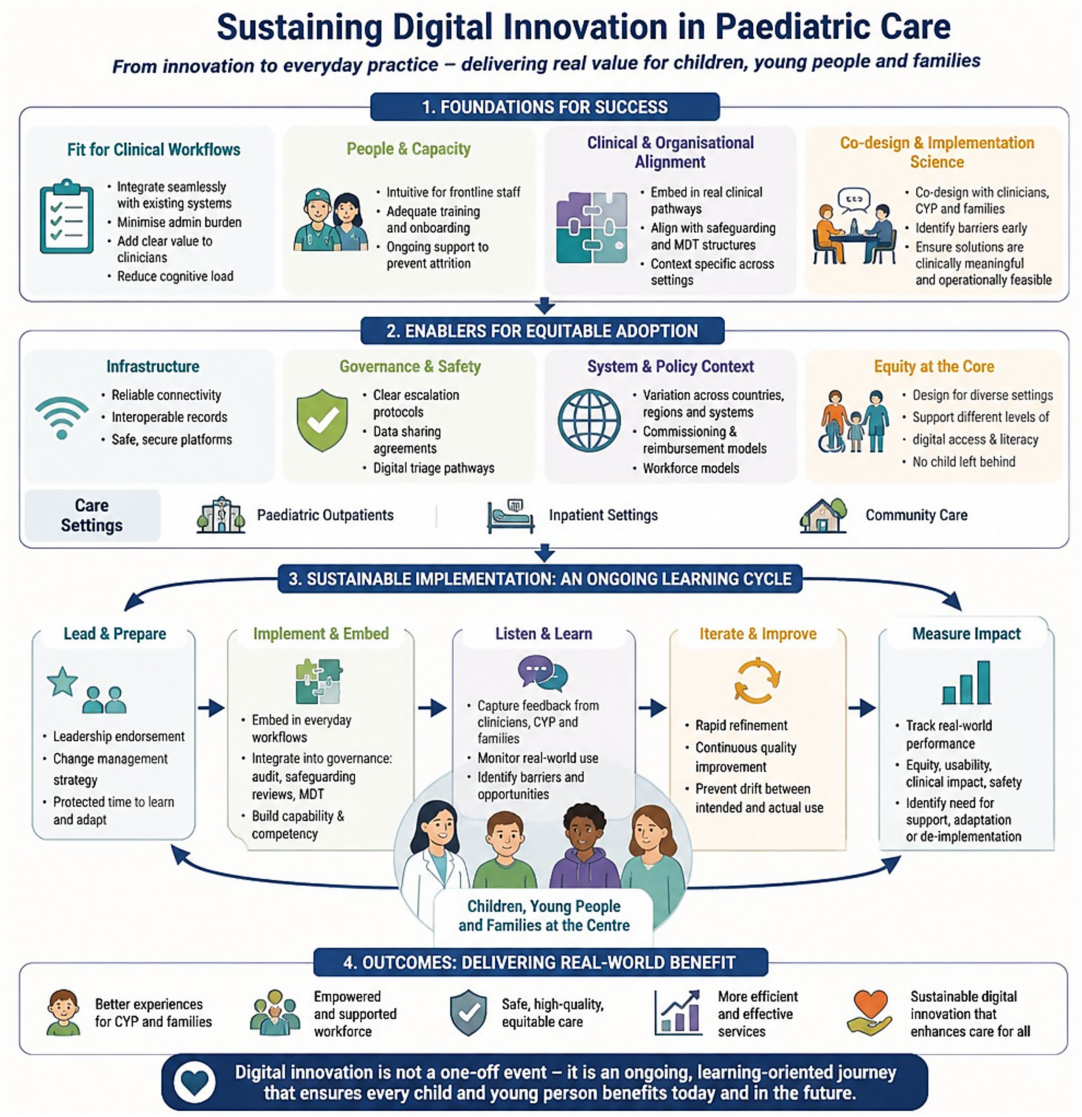
Key considerations for implementing digital innovations in paediatric clinical practice, summarising the organisational, infrastructural, and behavioural factors that determine whether digital tools can be successfully embedded into routine paediatric care.

Translating digital innovations into routine paediatric clinical practice requires careful attention to the realities of clinical workflows, workforce capacity, and organisational readiness. Figure 1 summarises the full framework outlined in Sections 10.1–10.4, bringing together the foundations for successful implementation, the enablers of equitable adoption, the ongoing learning cycle required for sustainable delivery, and the real-world outcomes that digital technologies can achieve in paediatric care.

### 10.1 Foundations for successful implementation of digital technologies

Effective adoption of digital technologies in paediatric services depends on creating the right organisational, technical, and behavioural conditions for change. New tools must integrate seamlessly with existing systems, minimise administrative burden, and provide clear value to clinicians, rather than adding parallel processes or increasing cognitive load. Successful implementation requires technologies that are intuitive for frontline staff, supported by adequate training and ongoing support to prevent attrition from technology use, and embedded within clinical pathways that reflect how care is delivered. This includes aligning digital tools with safeguarding processes, multidisciplinary team structures, and ensuring that they are context-specific in service delivery areas such as paediatric outpatients, inpatient settings, and community care. Embedding implementation science principles and co-design approaches with clinicians, patients and families helps identify practical, cultural, and workflow-related barriers early, ensuring that digital solutions are grounded in the realities of everyday care. This collaborative approach strengthens the clinical relevance of technologies, supports operational feasibility, and builds ownership among end users, whether they are clinicians, children, young people or parents. It also enhances feasibility and usability by ensuring that tools align with existing practices, reduce friction in care delivery, and respond to the needs and preferences of those who will ultimately rely on them. In doing so, it lays the foundations for sustained acceptance, adoption and meaningful impact.

Taken together, these elements highlight that successful digital transformation is not achieved through technology alone, but through the readiness of the system that surrounds it. Organisations must create the cultural, infrastructural and leadership conditions that include clear governance, protected time for staff to engage with new ways of working, and mechanisms for continuous learning and improvement. When digital technologies are introduced within an environment that values adaptation, supports staff through change, and prioritises the needs and experiences of children, young people, and families, they are far more likely to deliver sustained improvements in care. Establishing these foundations ensures that innovation is a coherent, long-term shift toward safer, more efficient and more responsive paediatric services.

### 10.2 Enablers for equitable adoption

Beyond usability and workflow alignment, organisations also need the infrastructure, governance, and policy frameworks that enable safe, equitable, and sustainable deployment of digital technologies. Reliable connectivity, interoperable records, and secure platforms form the backbone of effective digital care, ensuring that systems communicate seamlessly and data flows safely between services. Robust governance structures, including clear escalation protocols, data-sharing agreements, and defined digital triage pathways, are essential for maintaining patient safety and accountability. Implementation must also recognise variation across health-care systems, where differences in commissioning, reimbursement, and workforce models shape what is feasible in practice. Embedding these technologies within a supportive system and policy context ensures that adoption is aligned with national and regional priorities. Crucially, equity must remain at the core of digital transformation. Tools should be designed for diverse care settings, from paediatric outpatients and inpatient wards to community clinics, and must accommodate varying levels of digital access and literacy among families. Ensuring that no child is left behind requires deliberate strategies to bridge digital divides, support inclusion, and tailor solutions to local contexts. Ultimately, when infrastructure, governance, policy context, and equity are addressed together, digital transformation becomes embedded in the culture of care, supporting clinicians, empowering families, and improving outcomes for children across all settings. Sustained investment, cross-sector collaboration, and commitment to evaluation will ensure that these technologies evolve alongside clinical practice, maintaining trust, safety, and relevance. In doing so, digital health becomes a tool for efficiency and a catalyst for more inclusive, responsive, and compassionate paediatric services.

### 10.3 Sustainable implementation: an ongoing learning cycle

Sustained implementation depends on creating the organisational conditions that allow new tools to become part of everyday paediatric practice rather than short-lived pilots. This begins with leadership endorsement and a clear change-management strategy that recognises digital transformation as a cultural shift, not just a technical upgrade. Leaders must articulate a shared vision for how technology enhances care, allocate resources to support adoption, and model engagement with new systems. Protected time for staff to learn, adapt, and reflect is essential; without it, even well-designed tools risk being side-lined by competing clinical pressures. Building capability and confidence across multidisciplinary teams ensures that digital solutions are not confined to early adopters but become embedded in collective practice. This phase sets the foundation for sustainability by fostering trust, readiness, and shared ownership of innovation.

Once leadership and preparation are in place, technologies must be implemented and embedded within the clinical workflow. Integration into existing governance structures, such as audit cycles, safeguarding reviews, and multidisciplinary team meetings, helps normalise their use and maintain accountability for safety and effectiveness. Embedding digital tools in routine workflows also requires attention to interoperability, data security, and alignment with local service models. Implementation should be iterative, allowing teams to refine processes as they learn, and supported by structured training and competency frameworks that build long-term digital literacy. When digital systems become part of the same governance and quality-assurance mechanisms that underpin clinical care, they cease to be external add-ons and instead become integral to how paediatric services operate and improve.

A critical component of sustainable implementation is measuring impact in real-world settings, ensuring that digital tools deliver the outcomes they promise and remain aligned with clinical priorities. This requires a structured approach to evaluation that goes beyond initial deployment, incorporating routine monitoring of usability, workflow effects, safety signals, and clinical outcomes. Metrics should capture efficiency and performance but also equity of access, identifying whether certain groups of children, young people or families experience barriers or differential benefit. Continuous data collection enables organisations to detect unintended consequences early, such as increased workload, alert fatigue, or disparities in uptake, and to make informed decisions about where additional support, redesign or de-implementation may be needed. Embedding these measurement processes within existing quality-improvement and governance frameworks ensures that digital tools remain accountable, transparent, and responsive to the evolving needs of paediatric care.

### 10.4 Outcomes: delivering real-world impact

The goal of digital transformation in paediatric care is to deliver tangible, measurable improvements for CYP, families, and the workforce that supports them. When technologies are implemented thoughtfully and sustained through ongoing learning cycles, they create better experiences for CYP and families, simplifying access, improving communication, and enabling more personalised, coordinated care. Digital tools can reduce fragmentation between services, empower families to participate actively in decision-making, and enhance continuity across outpatient, inpatient, and community settings. Equally important is the creation of an empowered and supported workforce. Digital systems that are intuitive, interoperable, and embedded in clinical workflows free clinicians from administrative burden, allowing them to focus on direct patient care. When staff are trained, supported, and involved in co-design, technology becomes an enabler rather than an obstacle, strengthening professional confidence and collaboration across multidisciplinary teams.

Sustained digital adoption also contributes to safe, high-quality, and equitable care. Real-time data sharing, automated alerts, and integrated safeguarding pathways enhance clinical oversight and reduce risk. By embedding equity principles into design and evaluation, digital innovation ensures that children and families, regardless of geography, socioeconomic status, or digital literacy, receive consistent, high-quality care. Streamlined workflows, predictive analytics, and remote monitoring can reduce unnecessary hospital visits, improve early intervention, and support continuity of care across settings.

Ultimately, these outcomes collectively underpin sustainable digital innovation that enhances care for all. Sustainability arises when technology evolves alongside practice, guided by continuous feedback, evaluation, and adaptation. It ensures that digital tools remain relevant, trusted, and responsive to the changing needs of paediatric populations.

## 11 Future directions of paediatric digital health

The coming decade offers an unprecedented opportunity to reimagine paediatric healthcare through technologies that are intelligent, connected, personalised, and child-centred. Paediatric digital health is moving toward learning health systems. These are dynamic ecosystems in which data from clinical encounters, research studies, and real-world settings continuously inform improvement. These systems will depend on interoperable data infrastructure, real-time analytics, continuous model monitoring, and structured feedback loops between clinicians, CYP, and developers. Ultimately, data will be used to facilitate personalised and precision healthcare through the development of digital twins (183). These are virtual representations of individuals that simulate physiology, disease progression, and treatment response and could be a transformative tool in paediatrics. These models could support personalised care by modelling growth and developmental trajectories, simulating drug dosing and metabolism, predicting surgical and clinical outcomes, and optimising rehabilitation pathways (183–185). In line with this, scaling precision paediatrics will require population-level genomic screening, integration of multi-omic data into electronic health records, AI-enabled interpretation tools, equitable access to testing and counselling, and robust governance for genomic data. Future models will combine genomic information with digital biomarkers to generate dynamic, personalised risk profiles that evolve with the child, enabling earlier intervention and more tailored care pathways (23, 183, 185, 186). Furthermore, integrating multi-omic, imaging, and sensor-derived data will enable highly individualised simulations, though substantial validation, transparency, and ethical oversight will be essential before clinical deployment. Such models will need to demonstrate not only technical accuracy but also robustness across diverse paediatric populations in real-world settings, with clear explanations of how different data types contribute to predictions. Ensuring transparent model behaviour, independent validation, and appropriate governance mechanisms will be critical to prevent over-reliance on complex systems that may embed bias or produce misleading outputs. Only through rigorous evaluation and accountable oversight can personalised simulations be safely translated into routine paediatric care, but their future potential is significant, offering a pathway toward more anticipatory, personalised, and equitable models of care (186–188). Importantly, the advent of synthetic AI will, in the future, enable the generation of high-fidelity, privacy-preserving datasets that can enhance model training, reduce bias, and accelerate innovation without exposing real children to unnecessary data risks.

Next,-generation biosensors will ultimately play a future role in gathering physiological and pathophysiological data that may ultimately feed into the development of digital twins. Biosensors will be ultra-thin, flexible, and suitable for all ages and meet the needs of CYP with sensory issues (189–192). They are likely to integrate multimodal signals including biochemical, mechanical, and electrophysiological data while incorporating energy-harvesting capabilities that reduce the need for charging (193, 194). Future sensors are likely to assess environmental factors, capturing contextual exposures such as pollution, allergens, and noise (195). These innovations will support continuous monitoring of long-term conditions, early detection of deterioration, and personalised behavioural interventions, enabling more proactive and precise paediatric healthcare.

Digital mental health will continue to expand in response to rising demand and workforce shortages. Future developments include AI-supported early detection of crisis risk (196), personalised digital cognitive behavioural therapy (197, 198), and immersive VR-based exposure interventions (60, 61, 83, 84). Ensuring safety and effectiveness will require rigorous evaluation, strong safeguarding mechanisms, and cultural and developmental adaptation to meet the diverse needs of CYP.

### 11.1 Advancing the workforce and workplace

The paediatric workforce will undergo significant transformation as digital tools become increasingly embedded in care. AI-supported multidisciplinary team meetings, the emergence of digital care coordinators, and new roles focused on data stewardship, digital safeguarding, and data management will reshape how teams collaborate and make decisions, and how healthcare systems manage and utilise data at scale (199, 200). These developments will require comprehensive training programmes that build digital literacy, critical appraisal skills, and confidence in working alongside AI and other digital systems (200). At the same time, AI will augment clinical workflows by supporting triage, risk stratification, diagnostic decision-making, personalised treatment planning, automated documentation, and predictive resource allocation (23, 201). Crucially, AI must enhance clinical judgement, with future systems designed around transparent interfaces, clinician-in-the-loop oversight, and robust safety and accountability frameworks (23, 202, 203). Realising this vision will demand sustained investment in workforce development and the development of new professional standards to ensure that paediatric clinicians are equipped to deliver safe, effective, and ethically grounded digital care (25, 204–206).

### 11.2 Sustainable investment and market reform

Sustained and strategic investment will be essential to ensure that paediatric digital health innovation is scalable, equitable, and delivers impact. Dedicated funding pathways are needed to support early-stage development, evaluation, and implementation of child-specific technologies (207). Blended finance models combining public funding, philanthropic investment, and private capital may help to de-risk innovation and accelerate translation into practice (208). Additionally, public–private partnerships will play a critical role in fostering collaboration between industry, academia, and health systems, enabling shared expertise and more efficient development pipelines (10, 11, 16, 209). Furthermore, targeted incentives for paediatric-specific technologies, including regulatory flexibilities, innovation prizes, and procurement commitments, could further stimulate market activity. Such measures help counteract the structural disincentives that often deter investment in child-focused innovation, where smaller markets, fragmented commissioning routes, and higher evidentiary expectations can limit commercial viability. Strategic incentives can signal clear demand, reduce early-stage risk for developers, and accelerate translation from prototype to practice. Thus, embedding paediatric-specific innovation pathways, advanced procurement models, and targeted financial mechanisms within national policy could play a pivotal role in building a sustainable pipeline of high-quality digital tools designed specifically for CYP.

Market failures are common in paediatric innovation because the commercial incentives to develop child-specific technologies are often weak. Populations are smaller, regulatory pathways more complex, and returns on investment are less predictable. As a result, promising digital tools may never reach scale, or may only be available in well-resourced health systems. Mechanisms to address these market failures are therefore essential (124, 210). Pooled purchasing, whereby multiple hospitals, regions, or countries jointly procure paediatric technologies could create sufficient demand to make development commercially viable while reducing costs for individual sites. This will require willing and sustained collaboration at a national and international scale across healthcare systems (210). Coordinated commissioning ensures that health systems align on standards, reimbursement models, and evaluation requirements, reducing fragmentation and giving innovators a clearer route to adoption (211–213). For example, global innovation funds have the potential to provide early-stage capital for high-impact paediatric technologies that would otherwise struggle to attract investment, particularly those targeting rare diseases, low-income settings, or underserved populations (214). Together, these mechanisms help ensure that digital advances do not remain siloed or accessible only to wealthy institutions, but instead support equitable, scalable, and sustainable transformation across diverse paediatric healthcare systems.

Finally, demonstrating cost-effectiveness will be essential if paediatric digital health innovations are to achieve sustainable adoption across diverse health-care systems (215). Commissioners and policymakers increasingly require evidence that technologies deliver measurable efficiency gains, such as reduced emergency attendances, fewer avoidable admissions, streamlined clinical workflows, and improved adherence, while also improving health and wellbeing outcomes for CYP. Importantly, economic evaluation must account for the unique features of paediatric care, where benefits often accrue over the life course rather than within a single budget cycle. Approaches such as lifecycle economic modelling, cross-sector cost analysis, and equity-adjusted value assessments can help capture long-term returns and ensure that innovations do not disproportionately benefit already advantaged groups. Embedding cost-effectiveness requirements within procurement, reimbursement, and regulatory pathways will be critical to ensuring that paediatric technologies deliver real-world value in clinical settings, support system sustainability, and uphold the principle that no child is left behind.

## 12 Conclusion

As AI, biosensing, digital therapeutics, and precision medicine mature, the art of the possible will shift from reactive, episodic care toward proactive, personalised, and continuous models of support that adapt to each child’s developmental trajectory and lived context. Realising this vision will require sustained commitment to equity, rigorous governance, and meaningful participation of CYP, families, and communities. It will also depend on collaboration, interoperable infrastructure, and investment in innovation ecosystems that prioritise the needs of CYP rather than retrofitting adult-oriented solutions. If these conditions are met, digital health can help build a future in which every child benefits from timely, anticipatory, and compassionate care where technology amplifies human connection, strengthens clinical decision-making, and ensures that children’s rights, voices, and wellbeing remain at the centre of health system transformation.
